# The Role of Recombination in the Origin and Evolution of Alu Subfamilies

**DOI:** 10.1371/journal.pone.0064884

**Published:** 2013-06-04

**Authors:** Ana Teixeira-Silva, Raquel M. Silva, João Carneiro, António Amorim, Luísa Azevedo

**Affiliations:** 1 IPATIMUP-Institute of Molecular Pathology and Immunology of the University of Porto, Porto, Portugal; 2 FCUP-Faculty of Sciences, University of Porto, Porto, Portugal; Louisiana State University, United States of America

## Abstract

Alus are the most abundant and successful short interspersed nuclear elements found in primate genomes. In humans, they represent about 10% of the genome, although few are retrotransposition-competent and are clustered into subfamilies according to the source gene from which they evolved. Recombination between them can lead to genomic rearrangements of clinical and evolutionary significance. In this study, we have addressed the role of recombination in the origin of chimeric Alu source genes by the analysis of all known consensus sequences of human Alus. From the allelic diversity of Alu consensus sequences, validated in extant elements resulting from whole genome searches, distinct events of recombination were detected in the origin of particular subfamilies of AluS and AluY source genes. These results demonstrate that at least two subfamilies are likely to have emerged from ectopic Alu-Alu recombination, which stimulates further research regarding the potential of chimeric active Alus to punctuate the genome.

## Introduction

Alus are the most abundant and successful Short Interspersed Nuclear Elements (SINEs) and are exclusively found in primate genomes. In humans, they represent nearly 10% of the nuclear genome corresponding to over 1 million copies and a frequency of one insertion per 3 Kb [1], [2]. An Alu is about 300 bp long and is composed by two monomers with origin in the 7SL RNA gene [2] punctuated by several CpG doublets and attached to one another by a poly-A stretch. A second poly-A tail is present at the 3′end. Active Alus, also known as source or master genes, are those that are able to generate progeny by reverse transcription of an Alu RNA molecule that is inserted in novel genomic locations [3], [4]. The Alu retrotransposition rate in humans was estimated to be 1/21 births [5] which is a significant contribution of these elements to human diversity. Most Alus in the genome are inactive as retrotransposition ability is often impaired by truncation of 5′ bases, shortening of the poly-A tail, or other mutations that occur during, or sometimes after, genome integration [6].

Due to their abundance in the genome, high GC content (more than 60%), sequence identity (70%–100%) and embedded short sequences that are hotspots for recombination [7], [8] Alus are prone to crossover and gene conversion events. Whenever Alu-mediated recombination causes genomic rearrangements (deletions, inversions and duplications) involving gene-coding sequences, deleterious effects are expected [9]–[14]. Alu-mediated rearrangements can also play an important role in genome evolution when involved in structural differences between individual genomes [15]–[17], species [7], [18] or transcriptional diversity (reviewed in [19]), from which phenotypic fluctuations result.

Alus were first classified in distinct subfamilies that share specific (diagnostic) positions [20]. But, since events of substitution back mutation and recombination [21] are frequent, such criterion was later changed to a collection of Alus that had origin in the same source gene [22], although multiple source genes can contribute to the same subfamily [23], [24].

Previous studies have documented cases of Alu chimerism [7] as a source of intra-subfamily variability [25], Alu re-activation [26], and as a source for the emergence of new subfamilies in New World monkeys [27]. In line with this, we posed the pertinent question: did any human Alu subfamily emerged from a chimeric source gene resulting from Alu-Alu recombination? This work aims at searching such chimeric elements in humans. We started by creating a database of subfamily polymorphic sites. Focusing mainly in insertion/deletion (indel) markers and motivated by findings in whole genome searches that established the presence of both insertion and deletion alleles in the extant genome, we were able to detect two cases of recombination: (a) AluSx4 and, (b) one cluster of subfamilies that includes either AluYe2, AluYe4, or AluYe5, AluYe6 and AluYf5. Our work establishes that Alu-Alu recombination offers the genome new elements which are free to retrotranspose, evolve and play their role in the emergence of phenotypic novelties.

## Materials and Methods

### Collection of Human Consensus Alus

Sequences corresponding to consensus Alu were retrieved from the Repbase Update [28] database and from previous work [22], [29], [30]. Manual inspection of all sequences revealed cases with more than a single consensus sequence documented for a specific subfamily, as is the case of AluYa1 subfamily. To avoid arbitrary decision on choosing the exact sequence representing the subfamily, all sequences were included in the analyses and distinguished as, for instance, AluYa1_1 and AluYa1_2. The collection of human Alu consensus sequences is provided in Text S1.

### Database of Polymorphic Sites in Consensus Alus

**Figure 1:**
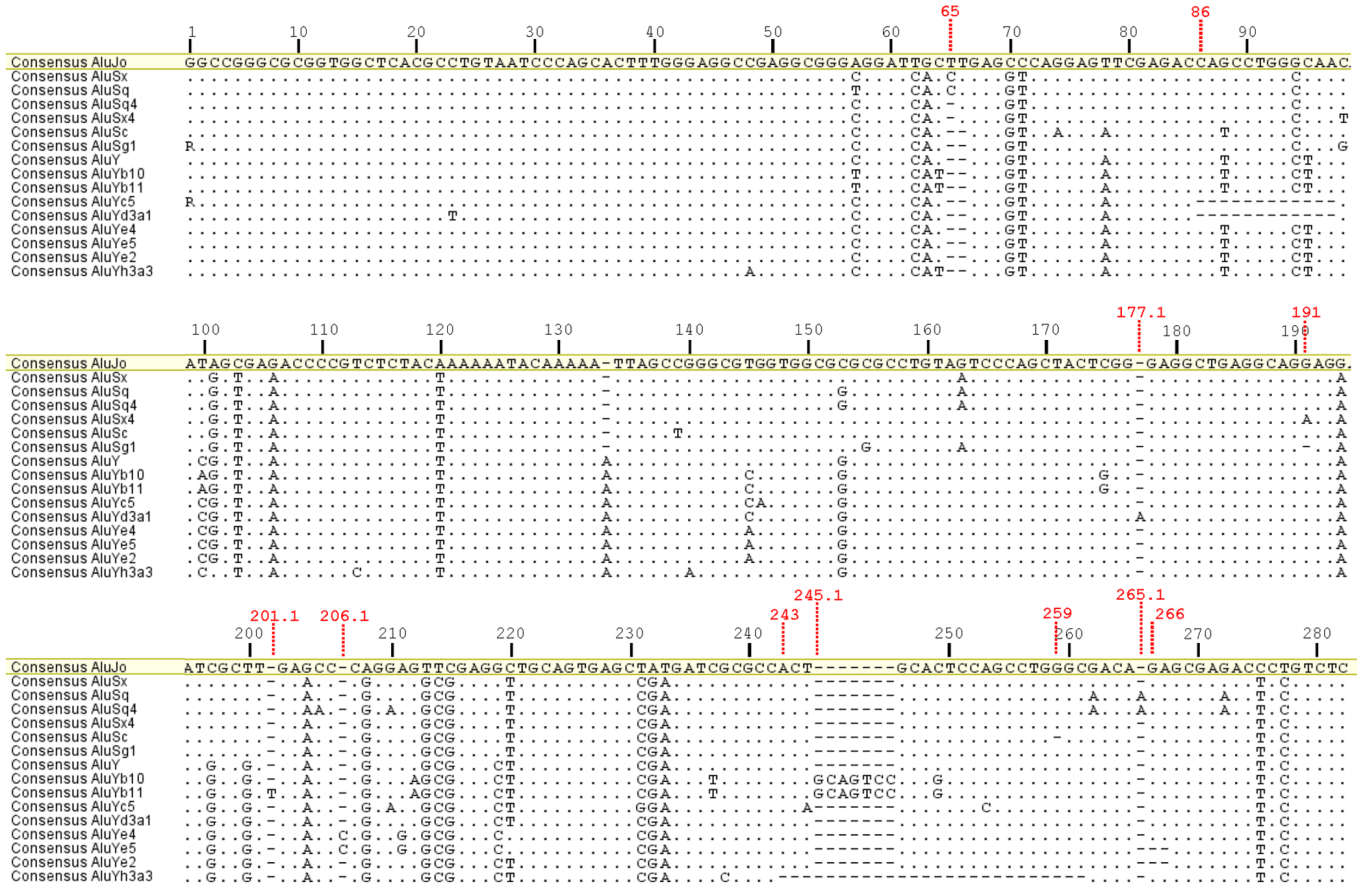
Alu consensus alignment and position numbering. Sequence Alignment of at least one representative of each haplotype defined by the 11 indel markers; node 1 is represented by two sequences: AluJo and AluSx. Position numbering was performed according to the reference AluJo. The first base of each indel is also indicated (red). Poly-A linker polymorphisms were disregarded. Dots represent identical bases and hyphens represent gaps (absent or deleted bases). R represents bases A or G according to the IUPAC code for nucleotide ambiguities.

The collection of consensus Alus was aligned in Geneious v5.4 [31] and poly-A tails were not considered (Text S2). The ancestral AluJo was set up as the reference sequence in our analyses and, consequently, position numbering was performed according to AluJo consensus sequence (Figure 1). Insertion and deletion polymorphisms (indels) were named as in the following example: a single-base deletion in position 65 is indicated as “65 del” and an insertion of an adenine after position 177 is indicated as “177.1 ins” (AluJo). Consensus Alus and polymorphic sites were then inputted into a database that provides all the information regarding the position and the distinct allelic forms of each polymorphism present in human consensus sequences. The database of Alu variability is accessible in Dataset S1.

### Whole Genome Search of Alu Indels

The presence of Alu indels in the extant human genome sequences was carried out using a Python script. The BioPython toolkit [32] was used to blast the NCBI human genome reference sequence (November 2012, Human Annotation Release 104) using an e-value threshold of 10^−5^ and allowing no gaps between the query and the subject sequence in order to prevent cross-contamination of each list with the counterpart allelic form. A total of 23 sequences (Table S1) were used as queries in the blast search. These sequences correspond to the 20 allelic forms of simple indels and a more complex pattern displayed in positions 65 and 66 with three allelic forms (65–66 ins: YT; 65 del: -T; 65–66 del: –). Each of these sequences was retrieved from a consensus Alu carrying the target allele (e.g., the 87–98 ins allele is represented by the AluY sequence whereas its counterpart, the 87–98 del allele, is represented by the AluYc5). The retrieved sequence hits were saved in fasta files and aligned in Geneious v5.4. The results were assembled in an excel format (Dataset S2) that holds a total of 144398 hits.

### Network Calculation using Indels

The Network 4611 software (http://www.fluxus-engineering.com/sharenet.htm) was used to cluster the entire collection of Alu sequences represented in the database. Allelic forma were converted in binary data (presence/absence) in the input file and only indel markers were used. Polymorphisms in poly-A linker and tail were not included. Each mutation site was equally weighted 10. All networks were calculated using the reduced median (RM) algorithm with the default parameters.

## Results

### Database of Polymorphic Sites for Consensus Alus

Most Alu copies inserted in the genome are inactive. This is especially evident in older subfamilies that no longer have active source genes due to a gradual accumulation of mutations. Analyses were performed using Alu consensus sequences, since it is important to consider the original sequence of each subfamily source gene. A consensus sequence is, by definition, a sequence that represents the very first source gene of a subfamily [33].

The collection of Alu consensus sequence retrieved from databases and related literature includes a total of 86 unique consensus sequences matching 73 distinct subfamilies. Of these, four correspond to the ancestral AluJ, 20 are documented as AluS sequences and 49 as AluY, the youngest family member [34]. Sequences were aligned for further comparison and AluJo was set as reference (Text S2). Position numbering was performed accordingly (Figure 1). This analysis revealed a total of 144 polymorphic positions (132 SNPs and 11 indels) that were combined into a database (Dataset S1) of Alu polymorphic variation. More than two alleles exist in 17 out of the 132 SNPs detected among Alu sequences, and in a single case (position 262 of AluJo) all four alleles were observed. The polymorphic indels show length sizes ranging from 1 to 19 bp (Dataset S1) and with the exception of positions 65 and 66, no size heterogeneity within the inserted/deleted sequence was observed.

### Whole Genome Search of Alu Indels

**Figure 2:**
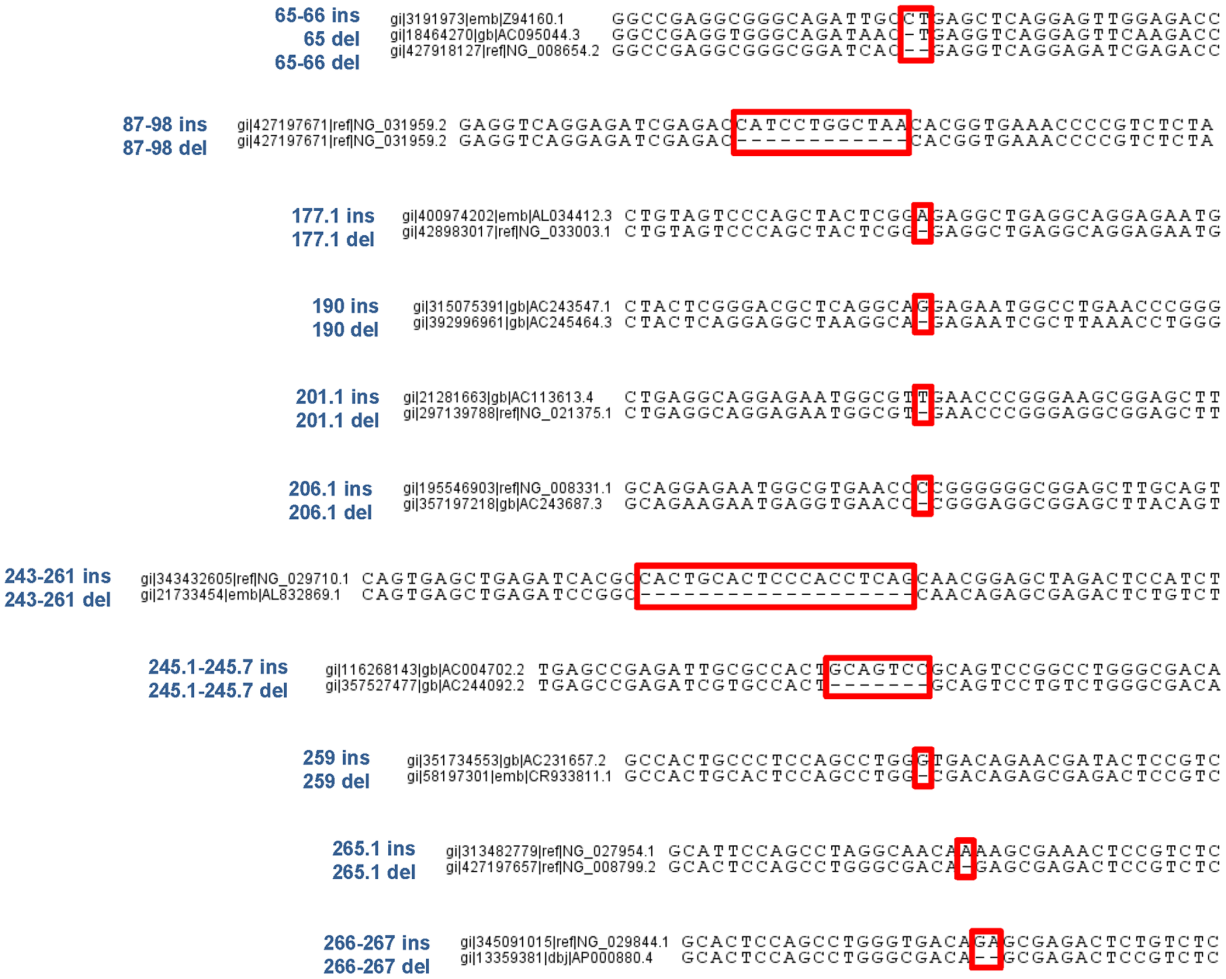
Sequence hits resulting from whole genome search of indel alleles carried by human consensus Alus. An example of a genomic sequence carrying each indel allele is given and aligned with the counterpart allele (left). The exact position of the indel is delimited. The complete list of results is provided in the Dataset S2.

Assuming that each consensus sequence evolved from pre-existent elements by mutation accumulation, we reconstructed the phylogenetic relationship between human Alu subfamilies using the available polymorphic information. Because many SNPs involve CpG dinucleotides and very few are subfamily-specific (see Dataset S1), we exploited the informativeness of indels discovered in the record of consensus sequences (Figure 1 and Dataset S1) to trace Alu lineages that date back 65 Myr [35]. To exclude the possibility that these insertions/deletions would result from errors or gaps during sequence reconstruction, a whole genome search was performed in the human reference sequence, as well as in nucleotide NCBI genome sequences using insertion and deletion alleles as queries. Examples of resulting sequence hits for each allele are presented in Figure 2 (and more detailed information is given in Dataset S2). No general conclusions can be made relative to allele frequencies, as this strategy was intended to identify highly similar sequences, discarding those which accumulated a significant number of mutations over time that are below the limits of detection.

### Evolutionary Clustering of Active Human Alus

**Figure 3:**
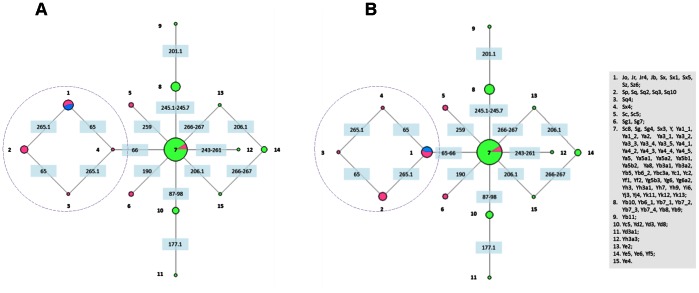
Clustering of Alu subfamilies using indel markers. The blue slice of node 1 represents the oldest subfamilies (AluJ). AluS elements are represented in pink and members of the young AluY are shown in green. Sites of mutational events are shown in blue boxes in the network’s branches. Networks A and B are the result of size heterogeneity in positions 65 and 66: (A) assuming that the three combinations resulted consecutively (65–66 ins –65 del –66 del) and (B) assuming that they were independent events (65–66 ins –65 del and 65–66 ins - 65–66 del). Networks A and B differ only in the right reticulation (circled) and the branch that connects it to node 7.

Once it was established that indel markers are not artifacts of sequence alignment at the time of consensus prediction, we used the haplotypic combination of indels to demonstrate the evolutionary relationships between Alu elements (Figure 3). As a result of size heterogeneity in positions 65 and 66 (65–66 ins, 65–66 del and 65 del), located in the left monomer, two networks were constructed: one assuming that the three combinations resulted consecutively (65–66 ins –65 del –66 del) (Figure 3A) and the other assuming that they were independent events (65–66 ins –65 del and 65–66 ins –65–66 del) (Figure 3B). Both analyses exhibited similar graphs, an indication that the origin of the mutational events does not alter the clustering inference.

**Figure 4:**
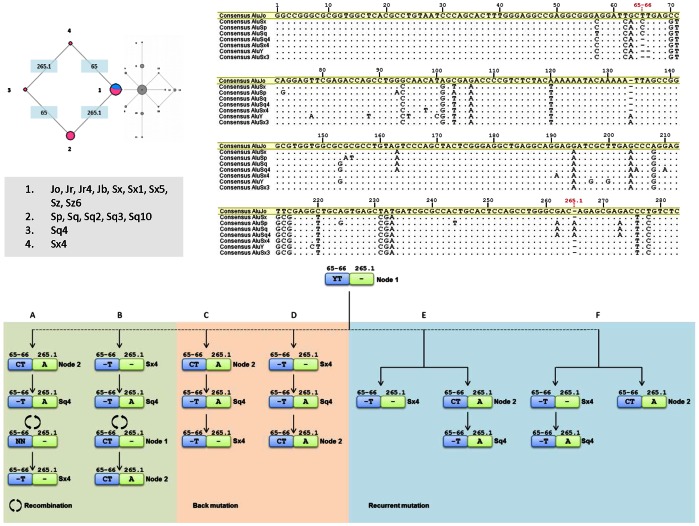
Alternative pathways for the origin of Alu subfamilies clustered in nodes 2, 3, and 4 of Figure 3. An alignment of at least a representative of each involved node is displayed, plus two representatives of node 7 (AluY and AluSx3). Alternative pathways are named A to F. A and B represent recombination events (green), C and D represent events of back mutation (orange) and E and F represent recurrent mutations (blue).

With the exception of two reticulations that clearly show alternative solutions to mutational events, both networks are well resolved revealing that most active genes originated from pre-existing sequences by mutation. The two reticulations that link nodes 1, 2, 3, 4 and 7, 13, 14, 15, may allude to events of Alu-Alu recombination and this hypothesis was further explored. In one of the cases (Figure 3, left reticulation), the Alu subfamilies represented in nodes 1, 2, 3 and 4 are distinguished by the haplotypic combination of 65–66 and 265.1 polymorphisms (Figure 4). Because positions 65 and 66 are deleted in the youngest AluY subfamily, and present in the old AluJo, the ancestral allele is 65–66 ins (Figure 3, node 1) [36]. Following the same rationale, the 265.1 ins is likely to be the youngest allele. Therefore, several alternative pathways were considered (Figure 4) based on the order of mutational events occurring in each monomer.

The most likely explanation for the emergence of these haplotypes is recombination (Figure 4, A and B). Path A illustrates the emergence of AluSx4 by recombination between an AluSq4 and an Alu lacking the 265.1 insertion, whereas path B shows the emergence of subfamilies on node 2 (AluSp, AluSq, AluSq2, AluSq3 and AluSq10) by recombination of an AluSq4 and an Alu from node 1. In-depth analyses of the consensus sequences of the subfamilies involved made it possible to discern the most parsimonious hypothesis: path A. AluSx4 differs from AluSq4 by the T98C substitution in the left monomer (Figure 4, alignment) and values of pairwise identity among the right monomers of all possible candidates to be donors (those not carrying the 265.1 ins) revealed that the most likely contributor was AluSx3 since both differ in a single site (G191A) (Figure 4) and share 99.3% of sequence identity. Both SNPs 98C and 191A are specific of AluSx4. Pathway B is less likely as it would require a minimum of ten extra mutational steps subsequently to the putative recombination between AluSq4 and elements of node 1. Although both pathways involve a recombination event, the one that requires less mutational steps is pathway A, which points to the origin of the AluSx4 subfamily through the recombination between an AluSq4 and Sx3 (Figure 3 and 4). If this is the case, the most likely representation of Alu evolution is shown in Figure 3B, that is the deletion at positions 65–66 had origin in the ancestral 65–66 ins allele.

Indels have a very low mutation rate, less than on tenth of SNP’s mutation rate [37]. Although less likely, events of indel back (Figure 4, C and D) or recurrent mutations (Figure 4, E or F) are also possible explanations for the emergence of the observed haplotypes. Concerning back mutation events, path C describes the emergence of AluSx4 by the deletion of base 265.1 in an AluSq4. In path D, the subfamilies of node 2 emerge from an AluSq4 by the re-insertion of a C in position 65. These paths are characterized by the succession of mutations in which the emergence of the ancestral allele is possible although extremely unlikely concerning an indel marker. Also, events of recurrent mutation are equally possible and equally unlikely. Path E illustrates the independent origin of AluSx4 and the elements of node 2, followed by the origin of AluSq4 through a deletion of base 65, while path F shows the independent insertion of base 265.1.

**Figure 5:**
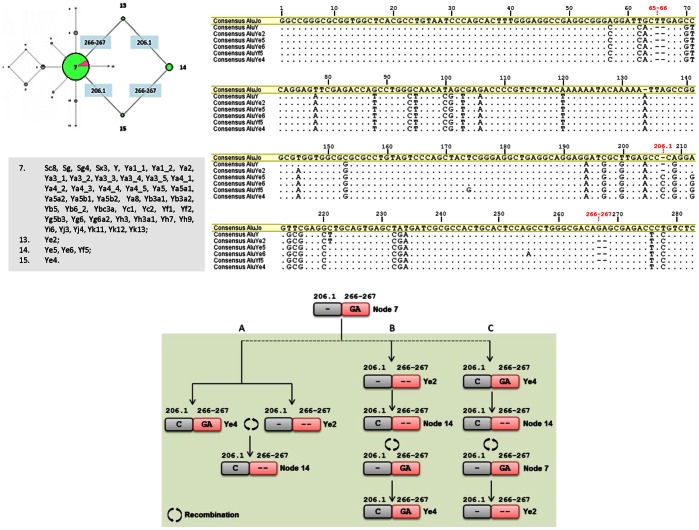
Alternative pathways for the origin of Alu subfamilies clustered in nodes 13, 14 and 15 of Figure 3. An alignment of at least one representative of each involved node is displayed. Alternative pathways are named A to C and represent recombination events (green).

The network reticulation on the right (Figure 3) has an even higher number of possible explanations for the appearance of the observed haplotypes (Figure 5). In this case, the key positions to establish the alternative mutational pathways are the 206.1 and 266–267.

Three pathways (Figure 5 A, B and C) imply an event of recombination. Regarding path A, assuming that AluYe4 and AluYe2 resulted from mutations in distinct lineages (206.1 ins and 266–267 del, respectively) of an ancestral sequence in node 7, and that a recombination event occurred between them, the ancestral of the subfamilies in node 14 (AluYe5, AluYe6 and AluYf5) was a recombinant Alu. With respect to pathway B, AluYe4 is a recombinant composed by a 5′part from a member of node 14 (AluYe5, AluYe6 or AluYf5) and a 3′ part from an element with the 266–267 ins allele from a member of nodes 7, 10 or 11. Lastly, pathway C describes AluYe2 as a recombinant between one of the elements from node 14 (AluYe5, AluYe6 or AluYf5) Alu and an element from node 7.

As with the previous example, the allelic configuration of these elements was analysed and combined with information provided by pairwise identity scores between the involved elements. These analyses did not reveal the most parsimonious hypothesis, as the identity scores between recombinant (chimeric) Alus and their corresponding parental elements reached about 100% in all cases, which is the result of the recent origin of the AluY subfamily [34]. Events of back and recurrent mutation (Figure S1) could also explain the existence of the haplotypes of these subfamilies; however, due to the recent advent (20 Mya) of the AluY clade [38], these hypotheses are even less likely to occur. Back and recurrent mutations are even rarer when considering indels longer that 1 bp, which is the case of indel 266–267. Furthermore, since the allele 206.1 del seems to be strongly associated with two SNPs (211A and 220T, Figure 1), events of back mutation would also have to occur in those two sites to result in the haplotypic combination observed in these subfamilies, which reinforces the unlikeliness of these events.

## Discussion

### Alu Master Genes can Originate through Recombination

Events of ectopic recombination among Alu elements are known to be associated with deleterious rearrangements [9]–[11], [13], [14], [39] and Alu chimerization [22], [25], [26], [40] as are for instance those reactivated by partial gene conversion involving the poly-A tail at the 3′end [26]. In this study, we searched for signs of recombination in known consensus sequences that represent the original source gene of each subfamily. Although predicted based on sequence homology, each consensus Alu must carry all the necessary elements to the retrotransposition process. Previous work tested 13 consensus Alus (AluJo, AluSx, AluY, AluYa5, AluYa5a2, AluYb8, AluYc1, AluYd8, AluYe5, AluYf2, AluYg6, AluYi6, AluYj4) and showed that all of them are able to retrotranspose, including the ancient AluJo [29].

We started by collecting all known consensus Alu sequences in humans and compiled them in a database that includes 86 sequences from 73 subfamilies and a total of 144 polymorphic positions (Dataset S1). Among the polymorphisms found, 11 are indels and were used to establish the historical relationship between the distinct subfamilies. The graphical clustering of all 73 Alu subfamilies revealed two distinct reticulations (Figure 3) that were analysed to evaluate all possible mutational and/or recombination events. After considering all possible pathways we could establish the role of Alu-Alu recombination in the origin of chimeric master genes, though it is not clear whereas the underlying mechanism was crossover or gene conversion. Our uncertainty in distinguishing between crossover and gene conversion is due to the lack of information on the flanking genomic region of the original master genes. Although gene conversion has been assumedly more frequent than crossover in Alu recombination [41]–[43], direct proof of gene conversion would only be possible if both recombination products were available [44].

### The Family Tree of Human Alus based on Polymorphic Information

**Figure 6:**
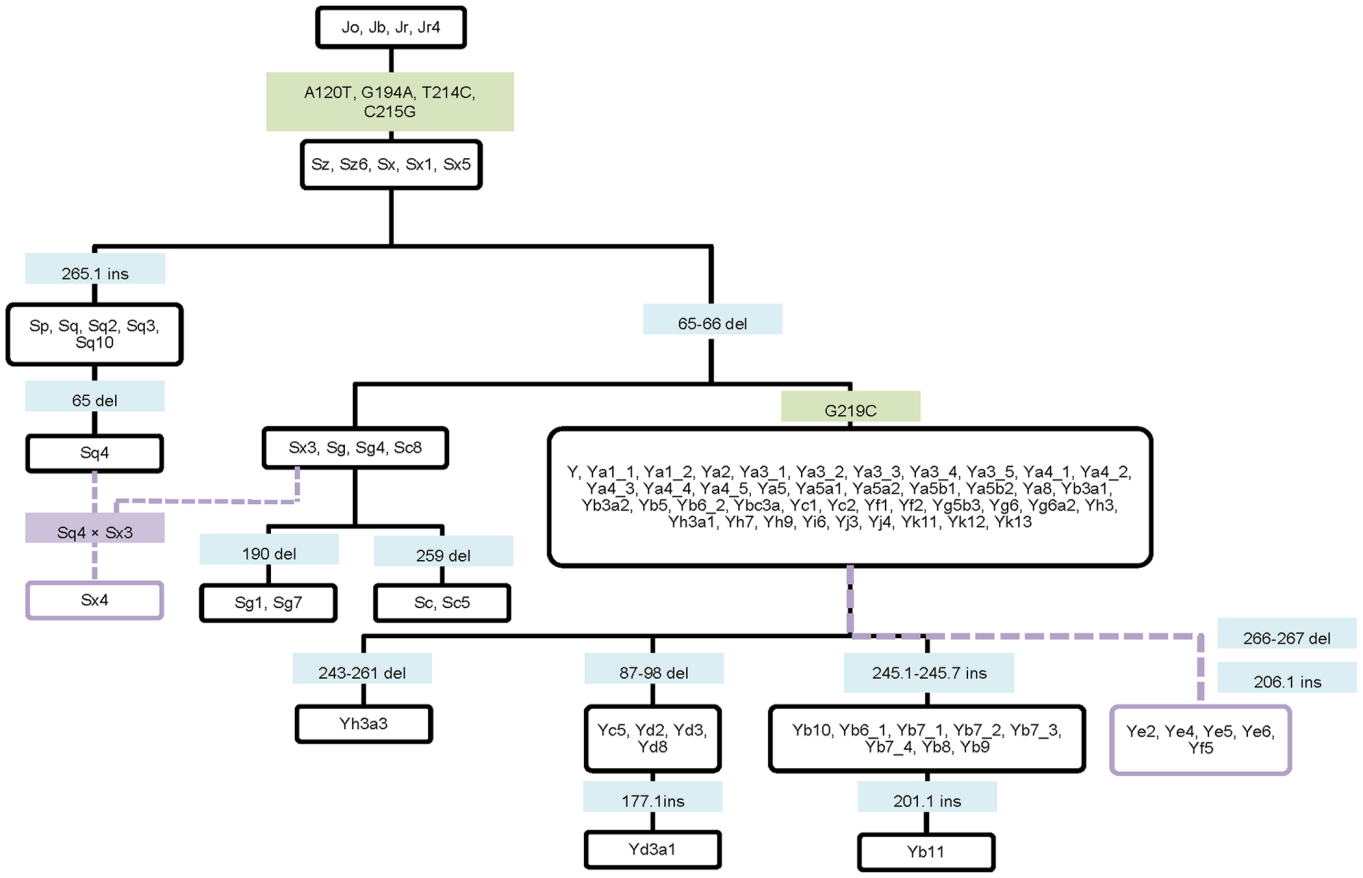
Evolution of human Alu subfamilies. Blue boxes correspond to indel markers, green boxes correspond to SNPs and purple boxes correspond to putative recombination events and recombinant (chimeric) subfamilies.

A general analysis of the information provided by both indels and SNPs allowed the distinction of Alu subfamilies according to informative positions (Figure 6). Despite the information provided by the combination of both marker types, large clusters incorporating a vast number of subfamilies, mainly in what refers to young AluY elements, are still observed. It is important to mention that although a high number of polymorphic positions were detected among Alu consensus sequences (Dataset S1), only A120T, G194A, T214C, C215G and G219C represent single occurrences in the history of Alu sequences that can be used as diagnostic positions (Figure 6).

Data presented in Figure 6 is relevant in many other aspects. There are cases in which subfamilies with more than one consensus sequence were clustered in distinct nodes, having different haplotypic combinations, as is the case of AluYb6. Such cases reveal that the boundaries of individualization of a subfamily are unclear. So, the questions we put forward are: (a) by how many mutational steps can a source gene differ from its parental gene and still be considered as a subfamily member and, the other way around, (b) how many mutations are necessary for an Alu sequence to be considered the founder of a new subfamily? Although we were able to detect two cases of recombination, or approach may have failed to detect additional cases of subfamilies that emerged by the same process. More data is needed in order to evaluate the complex role of ectopic recombination in the birth of chimeric Alu elements with retrotransposition ability, thus increasing genomic variability, creating new Alu insertions, and promoting further non-allelic homologous recombination.

## Supporting Information

Figure S1 **Additional alternative pathways for the origin of Alu subfamilies clustered in nodes 13, 14 and 15 of** **Figure 3** **.** Alternative pathways are named A to G. A, B and C represent recombination events (green), D and E represent events of back mutation (orange) and F and G represent recurrent mutations (blue).
(TIF)

Table S1 **Table of query sequences used in the whole genome search. “ins” defines the presence of extra nucleotides (red) and “del” their absence relative to AluJo.**
(DOC)

Text S1 **List of human Alu consensus sequences.**
(DOCX)

Text S2 **Complete alignment of human Alu consensus sequences.**
(TXT)

Dataset S1 **Database of all polymorphic positions detected in the complete list of human consensus Alus.** Position numbering was performed accordingly to AluJo. Major subfamily-specific mutations are coloured blue (sites 120, 194, 214 and 215) and green (site 219) and are specific of AluJ and AluY, respectively. Other subfamily-specific mutations are coloured grey.
(XLSX)

Dataset S2 **Human sequences that match each indel allele retrieved from whole genome searches.**
(XLS)

## References

[1] Lander ES, Linton LM, Birren B, Nusbaum C, Zody MC, et al (2001) Initial sequencing and analysis of the human genome. Nature 409: 860–921. 11237011 10.1038/35057062

[2] Ullu E, Tschudi C (1984) Alu sequences are processed 7SL RNA genes. Nature 312: 171–172. 6209580 10.1038/312171a0

[3] Rogers JH (1985) The origin and evolution of retroposons. International Review of Cytology-a Survey of Cell Biology 93: 187–279. 10.1016/s0074-7696(08)61375-3 2409043

[4] Weiner AM, Deininger PL, Efstratiadis A (1986) nonviral retroposons - genes, pseudogenes, and transposable elements generated by the reverse flow of genetic information. Annual Review of Biochemistry 55: 631–661. 10.1146/annurev.bi.55.070186.003215 2427017

[5] Xing J, Zhang Y, Han K, Salem AH, Sen SK, et al (2009) Mobile elements create structural variation: analysis of a complete human genome. Genome Res 19: 1516–1526. 19439515 10.1101/gr.091827.109 PMC2752133

[6] Comeaux MS, Roy-Engel AM, Hedges DJ, Deininger PL (2009) Diverse cis factors controlling Alu retrotransposition: What causes Alu elements to die? Genome Research 19: 545–555. 19273617 10.1101/gr.089789.108 PMC2665774

[7] Han K, Lee J, Meyer TJ, Wang J, Sen SK, et al (2007) Alu recombination-mediated structural deletions in the chimpanzee genome. PLoS Genet 3: 1939–1949. 17953488 10.1371/journal.pgen.0030184 PMC2041999

[8] Myers S, Freeman C, Auton A, Donnelly P, McVean G (2008) A common sequence motif associated with recombination hot spots and genome instability in humans. Nat Genet 40: 1124–1129. 19165926 10.1038/ng.213

[9] Pereira MC, Loureiro JL, Pinto-Basto J, Brandao E, Lopes AM, et al (2012) Alu elements mediate large SPG11 gene rearrangements: further spatacsin mutations. Genetics in medicine : official journal of the American College of Medical Genetics 14: 143–151. 22237444 10.1038/gim.2011.7

[10] Deininger PL, Batzer MA (1999) Alu repeats and human disease. Molecular Genetics and Metabolism 67: 183–193. 10381326 10.1006/mgme.1999.2864

[11] Dobrovolny R, Nazarenko I, Kim J, Doheny D, Desnick RJ (2011) Detection of Large Gene Rearrangements in X-linked Genes by Dosage Analysis: Identification of Novel alpha-Galactosidase A (GLA) Deletions Causing Fabry Disease. Human Mutation 32: 688–695. 21305660 10.1002/humu.21474

[12] Franke G, Bausch B, Hoffmann MM, Cybulla M, Wilhelm C, et al (2009) Alu-Alu recombination underlies the vast majority of large VHL germline deletions: Molecular characterization and genotype-phenotype correlations in VHL patients. Hum Mutat 30: 776–786. 19280651 10.1002/humu.20948

[13] Kuiper RP, Vissers LELM, Venkatachalam R, Bodmer D, Hoenselaar E, et al (2011) Recurrence and Variability of Germline EPCAM Deletions in Lynch Syndrome. Human Mutation 32: 407–414. 21309036 10.1002/humu.21446

[14] Quental R, Azevedo L, Rubio V, Diogo L, Amorim A (2009) Molecular mechanisms underlying large genomic deletions in ornithine transcarbamylase (OTC) gene. Clinical Genetics 75: 457–464. 19475717 10.1111/j.1399-0004.2009.01172.x

[15] Stewart C, Kural D, Stromberg MP, Walker JA, Konkel MK, et al (2011) A comprehensive map of mobile element insertion polymorphisms in humans. PLoS Genet 7: e1002236. 21876680 10.1371/journal.pgen.1002236 PMC3158055

[16] Stoneking M, Fontius JJ, Clifford SL, Soodyall H, Arcot SS, et al (1997) Alu insertion polymorphisms and human evolution: Evidence for a larger population size in Africa. Genome Research 7: 1061–1071. 9371742 10.1101/gr.7.11.1061 PMC310683

[17] Iskow RC, McCabe MT, Mills RE, Torene S, Pittard WS, et al (2010) Natural mutagenesis of human genomes by endogenous retrotransposons. Cell 141: 1253–1261. 20603005 10.1016/j.cell.2010.05.020 PMC2943760

[18] Sen SK, Han K, Wang J, Lee J, Wang H, et al (2006) Human genomic deletions mediated by recombination between Alu elements. American Journal of Human Genetics 79: 41–53. 16773564 10.1086/504600 PMC1474114

[19] Cowley M, Oakey RJ (2013) Transposable Elements Re-Wire and Fine-Tune the Transcriptome. PLoS Genet 9: e1003234. 23358118 10.1371/journal.pgen.1003234 PMC3554611

[20] Willard C, Nguyen HT, Schmid CW (1987) Existence of at least 3 distinct Alu subfamilies. Journal of Molecular Evolution 26: 180–186. 3129565 10.1007/BF02099850

[21] Zhi D (2007) Sequence correlation between neighboring Alu instances suggests post-retrotransposition sequence exchange due to Alu gene conversion. Gene 390: 117–121. 17134854 10.1016/j.gene.2006.09.030

[22] Styles P, Brookfield JFY (2007) Analysis of the features and source gene composition of the AluYg6 subfamily of human retrotransposons. Bmc Evolutionary Biology 7: 102. 17603915 10.1186/1471-2148-7-102 PMC1925064

[23] Matera AG, Hellmann U, Hintz MF, Schmid CW (1990) Recently transposed Alu repeats result from multiple source genes. Nucleic Acids Research 18: 6019–6023. 2172925 10.1093/nar/18.20.6019 PMC332399

[24] Cordaux R, Hedges DJ, Batzer MA (2004) Retrotransposition of Alu elements: how many sources? Trends in Genetics 20: 464–467. 15363897 10.1016/j.tig.2004.07.012

[25] Roy AM, Carroll ML, Nguyen SV, Salem AH, Oldridge M, et al (2000) Potential gene conversion and source genes for recently integrated Alu elements. Genome Research 10: 1485–1495. 11042148 10.1101/gr.152300

[26] Johanning K, Stevenson CA, Oyeniran OO, Gozal YM, Roy-Engel AM, et al (2003) Potential for retroposition by old Alu subfamilies. Journal of Molecular Evolution 56: 658–664. 12911029 10.1007/s00239-002-2433-y

[27] Ray DA, Batzer MA (2005) Tracking Alu evolution in New World primates. Bmc Evolutionary Biology 5. 10.1186/1471-2148-5-51 PMC1266357 16209711

[28] Jurka J, Kapitonov VV, Pavlicek A, Klonowski P, Kohany O, et al (2005) Repbase update, a database of eukaryotic repetitive elements. Cytogenetic and Genome Research 110: 462–467. 16093699 10.1159/000084979

[29] Bennett EA, Keller H, Mills RE, Schmidt S, Moran JV, et al (2008) Active Alu retrotransposons in the human genome. Genome Research 18: 1875–1883. 18836035 10.1101/gr.081737.108 PMC2593586

[30] Park ES, Huh JW, Kim TH, Kwak KD, Kim W, et al (2005) Analysis of newly identified low copy AluYj subfamily. Genes & Genetic Systems 80: 415–422. 16501310 10.1266/ggs.80.415

[31] Drummond A, Ashton B, Buxton S, Cheung M, Cooper A, et al.. (2011) Geneious 5.4 5.4 ed.

[32] Cock PJ, Antao T, Chang JT, Chapman BA, Cox CJ, et al (2009) Biopython: freely available Python tools for computational molecular biology and bioinformatics. Bioinformatics 25: 1422–1423. 19304878 10.1093/bioinformatics/btp163 PMC2682512

[33] Labuda D, Striker G (1989) Sequence conservation in Alu evolution. Nucleic Acids Research 17: 2477–2491. 2541408 10.1093/nar/17.7.2477 PMC317637

[34] Mighell AJ, Markham AF, Robinson PA (1997) Alu sequences. Febs Letters 417: 1–5. 9395063 10.1016/s0014-5793(97)01259-3

[35] Deininger PL, Daniels GR (1986) The recent evolution of mammalian repetitive DNA elements. Trends in Genetics 2: 76–80.

[36] Kapitonov V, Jurka J (1996) The age of Alu subfamilies. Journal of Molecular Evolution 42: 59–65. 8576965 10.1007/BF00163212

[37] Nachman MW, Crowell SL (2000) Estimate of the mutation rate per nucleotide in humans. Genetics 156: 297–304. 10978293 10.1093/genetics/156.1.297 PMC1461236

[38] Salem AH, Kilroy GE, Watkins WS, Jorde LB, Batzer MA (2003) Recently integrated Alu elements and human genomic diversity. Molecular Biology and Evolution 20: 1349–1361. 12777511 10.1093/molbev/msg150

[39] Batzer MA, Deininger PL (2002) Alu repeats and human genomic diversity. Nature Reviews Genetics 3: 370–379. 10.1038/nrg798 11988762

[40] Roy-Engel AM, Carroll ML, El-Sawy M, Salem AH, Garber RK, et al (2002) Non-traditional Alu evolution and primate genomic diversity. Journal of Molecular Biology 316: 1033–1040. 11884141 10.1006/jmbi.2001.5380

[41] McVean G (2010) What drives recombination hotspots to repeat DNA in humans? Philosophical Transactions of the Royal Society B-Biological Sciences 365: 1213–1218. 10.1098/rstb.2009.0299 PMC2871820 20308096

[42] Paigen K, Petkov P (2010) Mammalian recombination hot spots: properties, control and evolution. Nature Reviews Genetics 11: 221–233. 10.1038/nrg2712 PMC4389181 20168297

[43] Petrov DA (2001) Evolution of genome size: new approaches to an old problem. Trends in Genetics 17: 23–28. 11163918 10.1016/s0168-9525(00)02157-0

[44] Chen J-M, Cooper DN, Chuzhanova N, Ferec C, Patrinos GP (2007) Gene conversion: mechanisms, evolution and human disease. Nature Reviews Genetics 8: 762–775. 10.1038/nrg2193 17846636

