# Supplementary figures and images for: The Role of Recombination in the Origin and Evolution of Alu Subfamilies
Source: PLoS One. 2013 Jun 4;8(6):e64884. doi: 10.1371/journal.pone.0064884 (PMC3672193; doi:10.1371/journal.pone.0064884)

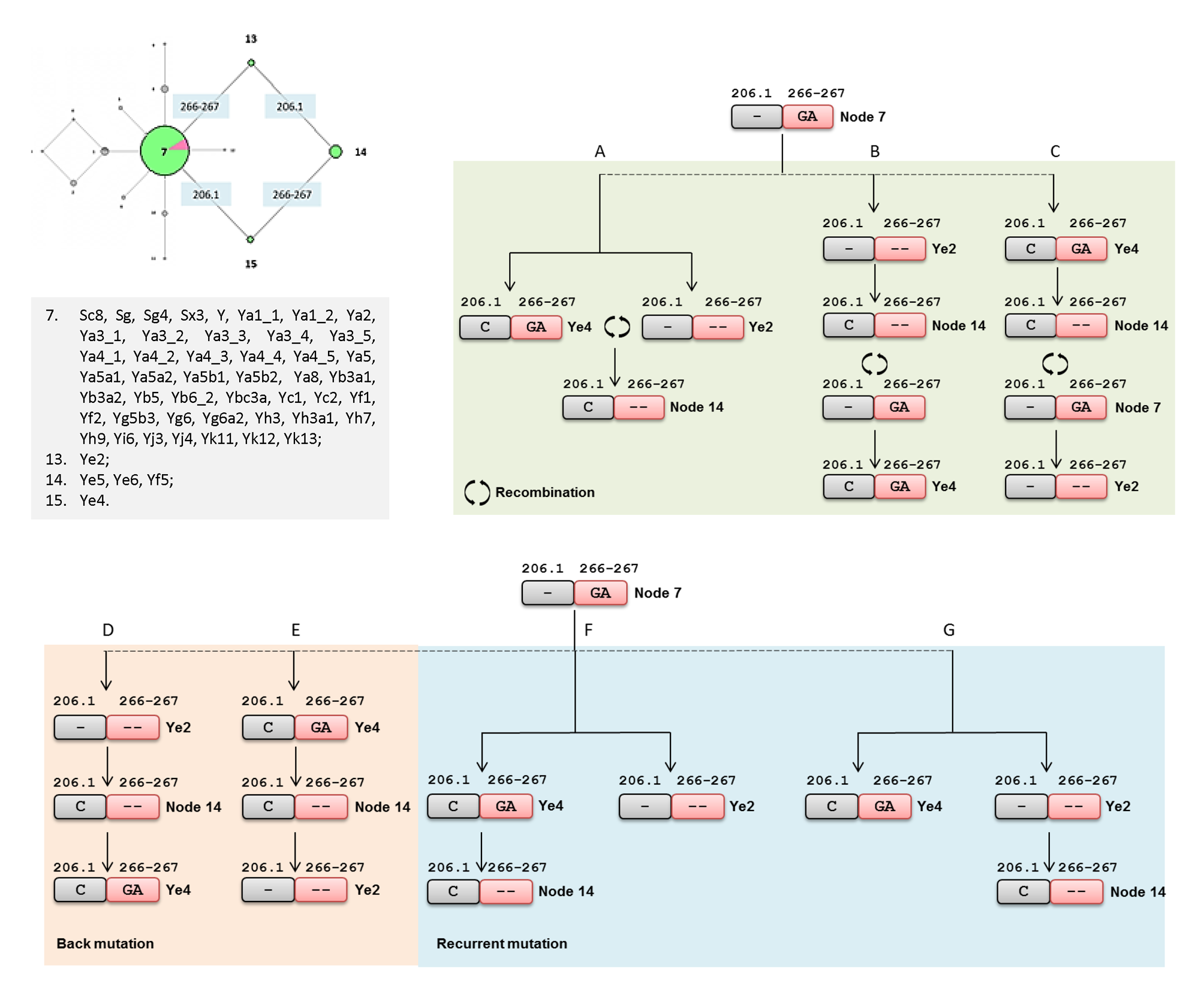

Supplement: Figure S1 — Additional alternative pathways for the origin of Alu subfamilies clustered in nodes 13, 14 and 15 of Figure 3 . Alternative pathways are named A to G. A, B and C represent recombination events (green), D and E represent events of back mutation (orange) and F and G represent recurrent mutations (blue). (TIF) [file pone.0064884.s001.tif]
