# Supplementary material for: The Role of Recombination in the Origin and Evolution of Alu Subfamilies
Source: PLoS One. 2013 Jun 4;8(6):e64884. doi: 10.1371/journal.pone.0064884 (PMC3672193; doi:10.1371/journal.pone.0064884)
Supplement: Table S1 — Table of query sequences used in the whole genome search. “ins” defines the presence of extra nucleotides (red) and “del” their absence relative to AluJo. (DOC) [file pone.0064884.s002.doc]

**Table of query sequences used in the whole genome search. “ins” defines the presence of extra nucleotides (red) and “del” their absence relative to AluJo.**

| **Allele designation** | **Sequence** | **Query sequence size** | **Query retrieved from consensus** | **Number of hits** |
| --- | --- | --- | --- | --- |
| 65-66 ins | GGCCGAGGCGGGCGGATCACCTGAGGTCAGGAGTTCGAGACC | 42 | Sx | 13,398 |
| 65 del | GGCCGAGGCGGGCGGATCACTGAGGTCAGGAGTTCGAGACC | 41 | Sx4 | 34 |
| 65-66 del | GGCCGAGGCGGGCGGATCACGAGGTCAGGAGATCGAGACC | 40 | Y | 5,516 |
| 87-98 ins | GAGGTCAGGAGATCGAGACCATCCTGGCTAACACGGTGAAACCCCGTCTCTA | 52 | Y | 16,979 |
| 87-98 del | GAGGTCAGGAGATCGAGACCACGGTGAAACCCCGTCTCTA | 40 | Yc5 | 604 |
| 177.1 ins | CTGTAGTCCCAGCTACTCGGAGAGGCTGAGGCAGGAGAATG | 41 | Yd3a1 | 592 |
| 177.1 del | CTGTAGTCCCAGCTACTCGGGAGGCTGAGGCAGGAGAATG | 40 | Y | 14,947 |
| 190 ins | CTACTCGGGAGGCTGAGGCAGGAGAATCGCTTGAACCCGGG | 41 | Sg | 17,024 |
| 190 del | CTACTCGGGAGGCTGAGGCAGAGAATCGCTTGAACCCGGG | 40 | Sg1 | 588 |
| 201.1 ins | CTGAGGCAGGAGAATGGCGTTGAACCCGGGAAGCGGAGCTT | 41 | Yb11 | 24 |
| 201.1 del | CTGAGGCAGGAGAATGGCGTGAACCCGGGAAGCGGAGCTT | 40 | Yb10 | 4,760 |
| 206.1 ins | GCAGGAGAATGGCGTGAACCCCGGGGGGCGGAGCCTGCAGT | 41 | Ye2 | 545 |
| 206.1 del | GCAGGAGAATGGCGTGAACCCGGGAGGCGGAGCTTGCAGT | 40 | Y | 5,622 |
| 243-261 ins | CAGTGAGCCGAGATCGCGCCACTGCACTCCAGCCTGGGCGACAGAGCGAGACTCCGTCT | 59 | Y | 19,304 |
| 243-261 del | CAGTGAGCCGAGATCCCGCCGACAGAGCGAGACTCCGTCT | 40 | Yh3a3 | 17 |
| 245.1-245.7 ins | TGAGCCGAGATTGCGCCACTGCAGTCCGCAGTCCAGCCTGGGCGACA | 47 | Yb11 | 576 |
| 245.1-245.7 del | TGAGCCGAGATCGCGCCACTGCACTCCAGCCTGGGCGACA | 40 | Y | 12,640 |
| 259 ins | GCCACTGCACTCCAGCCTGGGCGACAGAGCGAGACTCCGTC | 41 | Sg | 11,825 |
| 259 del | GCCACTGCACTCCAGCCTGGCGACAGAGCGAGACTCCGTC | 40 | Sc | 1,777 |
| 265 ins | GCACTCCAGCCTGGGCAACAAGAGCGAAACTCCGTCTC | 38 | Sq | 2,896 |
| 265 del | GCACTCCAGCCTGGGCGACAGAGCGAGACTCCGTCTC | 37 | SX | 7.075 |
| 266-267 ins | GCACTCCAGCCTGGGCGACAGAGCGAGACTCCGTCTC | 37 | Y | 7,075 |
| 266-267 del | GCACTCCAGCCTGGGCGACAGCGAGACTCCGTCTC | 35 | Ye2 | 580 |
