# Supplementary material for: The Role of Recombination in the Origin and Evolution of Alu Subfamilies
Source: PLoS One. 2013 Jun 4;8(6):e64884. doi: 10.1371/journal.pone.0064884 (PMC3672193; doi:10.1371/journal.pone.0064884)
Supplement: Text S1 — List of human Alu consensus sequences. (DOCX) [file pone.0064884.s003.docx]

**List of human Alu consensus sequences**

>AluJo

ggccgggcgcggtggctcacgcctgtaatcccagcactttgggaggccgaggcgggaggattgcttgagcccaggagttcgagaccagcctgggcaacatagcgagaccccgtctctacaaaaaatacaaaaattagccgggcgtggtggcgcgcgcctgtagtcccagctactcgggaggctgaggcaggaggatcgcttgagcccaggagttcgaggctgcagtgagctatgatcgcgccactgcactccagcctgggcgacagagcgagaccctgtctc

>AluJb

ggccgggcgcggtggctcacgcctgtaatcccagcactttgggaggccgaggcgggaggatcacttgagcccaggagttcgagaccagcctgggcaacatggtgaaaccccgtctctacaaaaaatacaaaaattagccgggcgtggtggcgcgcgcctgtagtcccagctactcgggaggctgaggcaggaggatcgcttgagcccgggaggtcgaggctgcagtgagccgtgatcgcgccactgcactccagcctgggcgacagagcgagaccctgtctc

>AluJr

ggccgggcgcggtggctcacgcctgtaatcccagcactttgggaggccgaggcgggaggatcgcttgaggccaggagttcgagaccagcctgggcaacatagcgagaccccgtctctacaaaaaatataaaaattagccgggcgtggtggcgcgcgcctgtagtcccagctactcgggaggctgaggcgggaggatcgcttgagcccaggagttcgaggctgcagtgagctatgatcgcgccactgcactccagcctgggcgacagagcgagaccctgtctc

>AluJr4

ggccgggcgcggtggctcacgcctgtaatcccagcactttgggaggccgaggcgggaggatcgcttgaggccaggagttcgagaccagcctgggcaacatagcgagaccccgtctctacaaaaaatntaaaaattagccgggcgtggtggcgcgcgcctgtagtcctagctactcgggaggctgaggcaggaggatcgcttgagcccaggagttcgaggttacagtgagctatgatcgcgccactgcactccagcctgggcgacagagcgagaccctgtctc

>AluSc

Ggccgggcgcggtggctcacgcctgtaatcccagcactttgggaggccgaggcgggcggatcacgaggtcaagagatcgagaccatcctggccaacatggtgaaaccccgtctctactaaaaatacaaaaattagctgggcgtggtggcgcgcgcctgtagtcccagctactcgggaggctgaggcaggagaatcgcttgaacccgggaggcggaggttgcagtgagccgagatcgcgccactgcactccagcctggcgacagagcgagactccgtctc

>AluSc5

Ggccgggcgcggtggctcacgcctgtaatcccagcactttgggaggccgaggcgggcggatcacgaggtcaggagatcgagaccatcctggccaacatggtgaaaccccgtctctactaaaaatacaaaaattagctgggcgtggtggcgcgtgcctgtaatcccagctactcgggaggctgaggcaggagaatcgcttgaaccagggagtcggaggttgcagtgagccgagatcgcgccactgcactccagcctggcgacagagcgagactccgtctc

>AluSc8

Ggccgggcgcggtggctcacgcctgtaatcccagcactttgggaggccgaggcgggcggatcacgaggtcaggagatcgagaccatcctggctaacacggtgaaaccccgtctctactaaaaatacaaaaaattagccgggcgtggtggcacgcgcctgtagtcccagctactcgggaggctgaggcaggagaatcgcttgaacccgggaggcggaggttgcagtgagccgagatcgcgccactgcactccagcctgggcgacagagcgagactccgtctc

>AluSg

Ggccgggcgcggtggctcacgcctgtaatcccagcactttgggaggccgaggcgggcggatcacgaggtcaggagttcgagaccagcctggccaacatggtgaaaccccgtctctactaaaaatacaaaaattagccgggcgtggtggcgcgcgcctgtaatcccagctactcgggaggctgaggcaggagaatcgcttgaacccgggaggcggaggttgcagtgagccgagatcgcgccactgcactccagcctgggcgacagagcgagactccgtctc

>AluSg1

Rgccgggcgcggtggctcacgcctgtaatcccagcactttgggaggccgaggcgggcggatcacgaggtcaggagttcgagaccagcctggccaagatggtgaaaccccgtctctactaaaaatacaaaaattagccgggcgtggtggcgcgggcctgtaatcccagctactcgggaggctgaggcagagaatcgcttgaacccgggaggcggaggttgcagtgagccgagatcgcgccactgcactccagcctgggcgacagagcgagactccgtctc

>AluSg4

Ggccgggcgcggtggctcacgcctgtaatcccagcactttgggaggccgaggcgggtggatcacgaggtcaggagttcgagaccagcctggccaagatggtgaaaccccgtctctactaaaaatacaaaaattagccgggcgcggtggcgggcgcctgtaatcccagctactcgggaggctgaggcaggagaatcgcttgaacccgggaggcggaggttgcagtgagccgagatcgcgccactgcactccagcctgggcgacagagcgagactccgtctc

>AluSg7

Ggccgggcgcggtggctcacgcctgtaatcccagcactttgggaggccgaggcgggtggatcacgaggtcaggagttcaagaccagcctggccaagatggtgaaaccccgtctctactaaaaatacaaaaattagccgggcgtggtggcgggcgcctgtaatcccagctactcgggaggctgaggcagagaattgcttgaacccgggaggcggaggttgcagtgagccgagatcgcgccactgcactccagcctgggcgacagagcgagactccgtctc

>AluSp

Ggccgggcgcggtggctcacgcctgtaatcccagcactttgggaggccgaggcgggcggatcacctgaggtcgggagttcgagaccagcctgaccaacatggagaaaccccgtctctactaaaaatacaaaaattagccgggcgtggtggcgcatgcctgtaatcccagctactcgggaggctgaggcaggagaatcgcttgaacccgggaggcggaggttgcggtgagccgagatcgcgccattgcactccagcctgggcaacaagagcgaaactccgtctc

>AluSq

Ggccgggcgcggtggctcacgcctgtaatcccagcactttgggaggccgaggcgggtggatcacctgaggtcaggagttcgagaccagcctggccaacatggtgaaaccccgtctctactaaaaatacaaaaattagccgggcgtggtggcgggcgcctgtaatcccagctactcgggaggctgaggcaggagaatcgcttgaacccgggaggcggaggttgcagtgagccgagatcgcgccactgcactccagcctgggcaacaagagcgaaactccgtctc

>AluSq10

Ggccgggcgcggtggctcacgcctgtaatcccagcactttgggaggccgaggcgggtggatcacctgaggtcaggagttctagaccagcctggccaacatggtgaaaccccgtctctactaaaaatacaaaaattagccgggcgtggtggcaggcgcctgtaatcccagctactcggggggccgaggcaggagaatcgcttgaacccgggaggcggaggttgcagtgagccgagatcgcgccatcgcactccagcctgggggacaagagcgagacttcgtctc

>AluSq2

Ggccgggcgcggtggctcacgcctgtaatcccagcactttgggaggccgaggcgggcggatcacctgaggtcaggagttcgagaccagcctggccaacatggtgaaaccccgtctctactaaaaatacaaaaattagccgggcgtggtggcgggcgcctgtaatcccagctactcgggaggctgaggcaggagaatcgcttgaacccgggaggcggaggttgcagtgagccgagatcgcgccattgcactccagcctgggcgacaagagcgaaactccgtctc

>AluSq3

GgccgggcgcggtggctcacgcctgtaatcccagcactttgggaggccgaggcgggcggatcacctgaggTcaggagttcgagaccagcctggccaacatggtgaaaccccgtctctactaaaaatacaaaaattagccgggcgtggtggcaggcgcctgtaatcccagctactcgggaggctgaggcaggagaatcgcttgaacccgggaggcggaggttgcagtgagccgagatcgcgccattgcactccagcctgggggacaagagcgagacttcgtctc

>AluSq4

Ggccgggcgcggtggctcacgcctgtaatcccagcactttgggaggccgaggcgggcggatcactgaggtcaggagttcgagaccagcctggccaacatggtgaaaccccgtctctactaaaaatacaaaaattagccgggcgtggtggcgggcgcctgtaatcccagctactcgggaggctgaggcaggagaatcgcttgaaaccggaaggcggaggttgcagtgagccgagatcgcgccactgcactccagcctgggcaacaagagcgaaactccgtctc

>AluSx

Ggccgggcgcggtggctcacgcctgtaatcccagcactttgggaggccgaggcgggcggatcacctgaggtcaggagttcgagaccagcctggccaacatggtgaaaccccgtctctactaaaaatacaaaaattagccgggcgtggtggcgcgcgcctgtaatcccagctactcgggaggctgaggcaggagaatcgcttgaacccgggaggcggaggttgcagtgagccgagatcgcgccactgcactccagcctgggcgacagagcgagactccgtctc

>AluSx1

ggccgggcgcggtggctcacgcctgtaatcccagcactttgggaggccgaggcgggcggatcacctgaggtcaggagttcgagaccagcctggccaacatggtgaaaccccgtctctactaaaaatacaaaaattagccgggcgtggtggcgggcgcctgtaatcccagctactcgggaggctgaggcaggagaatcgcttgaacccgggaggcggaggttgcagtgagccgagatcgcgccactgcactccagcctgggcgacagagcgagactccgtctc

>AluSx3

Ggccgggcgcggtggctcacgcctgtaatcccagcactttgggaggccgaggcgggcggatcacgaggtcaggagttcgagaccagcctggccaacatggtgaaaccccgtctctactaaaaatacaaaaaattagccgggcgtggtggcgcgcgcctgtagtcccagctactcgggaggctgaggcaggagaatcgcttgaacccgggaggcggaggttgcagtgagccgagatcgcgccactgcactccagcctgggcgacagagcgagactccgtctc

>AluSx4

Ggccgggcgcggtggctcacgcctgtaatcccagcactttgggaggccgaggcgggcggatcactgaggtcaggagttcgagaccagcctggccaatatggtgaaaccccgtctctactaaaaatacaaaaattagccgggcgtggtggcgcgcgcctgtagtcccagctactcgggaggctgaggcagaagaatcgcttgaacccgggaggcggaggttgcagtgagccgagatcgcgccactgcactccagcctgggcgacagagcgagactccgtctc

>AluSz

Ggccgggcgcggtggctcacgcctgtaatcccagcactttgggaggccgaggcgggcggatcacttgaggtcaggagttcgagaccagcctggccaacatggtgaaaccccgtctctactaaaaatacaaaaattagccgggcgtggtggcgcgcgcctgtaatcccagctactcgggaggctgaggcaggagaatcgcttgaacccgggaggcggaggttgcagtgagccgagatcgcgccactgcactccagcctgggcgacagagcgagactccgtctc

>AluSz6

Ggccgggcgcggtggctcacgcctgtaatcccagcactttgggaggccgaggcgggcggatcacttgaggccaggagttcgagaccagcctggccaacatggcaaaaccccgtctctactaaaaatacaaaaattagccgggcgtggtggcgcgcgcctgtaatcccagctactcgggaggctgaggcacgagaatcgcttgaacccgggaggcggaggttgcagtgagccgagatcgcgccactgcactccagcctgggcgacagagcgagactctgtctc

>AluY

Ggccgggcgcggtggctcacgcctgtaatcccagcactttgggaggccgaggcgggcggatcacgaggtcaggagatcgagaccatcctggctaacacggtgaaaccccgtctctactaaaaatacaaaaaattagccgggcgtggtggcgggcgcctgtagtcccagctactcgggaggctgaggcaggagaatggcgtgaacccgggaggcggagcttgcagtgagccgagatcgcgccactgcactccagcctgggcgacagagcgagactccgtctc

>AluYa5

Ggccgggcgcggtggctcacgcctgtaatcccagcactttgggaggccgaggcgggcggatcacgaggtcaggagatcgagaccatcccggctaaaacggtgaaaccccgtctctactaaaaatacaaaaaattagccgggcgtagtggcgggcgcctgtagtcccagctacttgggaggctgaggcaggagaatggcgtgaacccgggaggcggagcttgcagtgagccgagatcccgccactgcactccagcctgggcgacagagcgagactccgtctc

>AluYa8

Ggccgggcgcggtggctcacgcctgtaatcccagcactttgggaggccgaggcgggcggatcacgaggtcaggagatcgagaccatcccggctaaaacggtgaaaccccgtctctactaaaactacaaaaaatagccgggcgtagtggcgggcgcctgtagtcctagctacttgggaggctgaggcaggagaatggcgtgaacccgggaggcggagcttgcagtgagccgagatcccgccactgcactccagcctgggcgacagagcgagactccgtctc

>AluYb3a1

Ggccgggcgcggtggctcacgcctgtaatcccagcactttgggaggccgaggcgggtggatcacgaggtcaggagatcgagaccatcctggctaacacggtgaaaccccgtctctactaaaaatacaaaaaattagccgggcgtggtggcgggcgcctgtagtcccagctactcgggaggctgaggcaggagaatggcgtgaacccgggaggcggagcttgcagtgagccgagattgcgccactgcagtccagcctgggcgacagagcgagactccgtctc

>AluYb3a2

Ggccgggcgcggtggctcacgcctgtaatcccagcactttgggaggccgaggcgggcggatcacgaggtcaggagatcgagaccatcctggctaacacggtgaaaccccgtctctactaaaaatacaaaaaattagccgggcgcggtggcgggcgcctgtagtcccagctactcgggaggctgaggcaggagaatggcgtgaacccgggaggcggagcttgcagtgagccgagatagcgccactgcagtccggcctgggcgaaagagcgagactccgtctc

>AluYb8

ggccgggcgcggtggctcacgcctgtaatcccagcactttgggaggccgaggcgggtggatcatgaggtcaggagatcgagaccatcctggctaacaaggtgaaaccccgtctctactaaaaatacaaaaaattagccgggcgcggtggcgggcgcctgtagtcccagctactcgggaggctgaggcaggagaatggcgtgaacccgggaagcggagcttgcagtgagccgagattgcgccactgcagtccgcagtccggcctgggcgacagagcgagactccgtctc

>AluYb9

ggccgggcgcggtggctcacgcctgtaatcccagcactttgggaggccgaggcgggtggatcatgaggtcaggagatcgagaccatcctggctaacaaggtgaaaccccgtctctactaaaaatacaaaaaattagccgggcgcggtggcgggcgcctgtagtcccagctactggggaggctgaggcaggagaatggcgtgaacccgggaagcggagcttgcagtgagccgagattgcgccactgcagtccgcagtccggcctgggcgacagagcgagactccgtctc

>AluYbc3a

ggccgggcgcggtggctcacgcctgtaatcccagcactttgggaggccgaggcgggcggatcacgaggtcaggagatcgagaccatcctggctaacacggtgaaaccccgtctctactaaaaatacaaaaaaattagccgggcgtggtagcgggcgcctgtagtcccagctactcgggaggctgaggcaggagaatggcgtgaacccgggaggcggagcttgcagtgagccaagatagcgccactgcagtccagcctgggcgaaagagcgagactccgtctc

>AluYc1

ggccgggcgcggtggctcacgcctgtaatcccagcactttgggaggccgaggcgggcggatcacgaggtcaggagatcgagaccatcctggctaacacggtgaaaccccgtctctactaaaaatacaaaaaattagccgggcgtggtagcgggcgcctgtagtcccagctactcgggaggctgaggcaggagaatggcgtgaacccgggaggcggagcttgcagtgagccgagatcgcgccactgcactccagcctgggcgacagagcgagactccgtctc

>AluYc2

ggccgggcgcggtggctcacgcctgtaatcccagcactttgggaggccgaggcgggcggatcacgaggtcaggagatcgagaccatcctggctaacaaggtgaaaccccgtctctactaaaaatacaaaaaattagccgggcgtggtagcgggcgcctgtagtcccagctactcgggaggctgaggcaggagaatggcgtgaacccgggaggcggagcttgcagtgagccgagatcgcgccactgcactccagcctgggcgacagagcgagactccgtctc

>AluYc5

Rgccgggcgcggtggctcacgcctgtaatcccagcactttgggaggccgaggcgggcggatcacgaggtcaggagatcgagaccacggtgaaaccccgtctctactaaaaatacaaaaaattagccgggcgcagtggcgggcgcctgtagtcccagctactcgggaggctgaggcaggagaatggcgtgaacccggaaggcggagcttgcagtgagcggagatcgcgccacagcactcccgcctgggcgacagagcgagactccgtctc

>AluYd2

ggccgggcgcggtggctcacgcctgtaatcccagcactttgggaggccgaggcgggcggatcacgaggtcaggagatcgagaccacggtgaaaccccgtctctactaaaaatacaaaaaattagccgggcgcggtggcgggcgcctgtagtcccagctactcgggaggctgaggcaggagaatggcgtgaacccgggaggcggagcttgcagtgagccgagatcgcgccactgcactccagcctgggcgacagagcgagactccgtctc

>AluYd3

ggccgggcgcggtggctcacgcttgtaatcccagcactttgggaggccgaggcgggcggatcacgaggtcaggagatcgagaccacggtgaaaccccgtctctactaaaaatacaaaaaattagccgggcgcggtggcgggcgcctgtagtcccagctactcgggaggctgaggcaggagaatggcgtgaacccgggaggcggagcttgcagtgagccgagatcgcgccactgcactccagcctgggcgacagagcgagactccgtctc

>AluYd3a1

ggccgggcgcggtggctcacgcttgtaatcccagcactttgggaggccgaggcgggcggatcacgaggtcaggagatcgagaccacggtgaaaccccgtctctactaaaaatacaaaaaattagccgggcgcggtggcgggcgcctgtagtcccagctactcggagaggctgaggcaggagaatggcgtgaacccgggaggcggagcttgcagtgagccgagatcgcgccactgcactccagcctgggcgacagagcgagactccgtctc

>AluYd8

ggccgggcgcggtggctcacgcctgtaatcccagcactttgggaggccgaggcgggcggatcacgaggtcaggagatcgagaccacggtgaaaccccgtctctactaaaaatacaaaaaattagccgggcgcagtggcgggcgcctgtagtcccagctactcgggaggctgaggcaggagaatggcgtgaacccggaaggcggagcttgcagtgagcggagatcgcgccacagcactcccgcctgggcgacagaacgagactccgtctc

>AluYe2

ggccgggcgcggtggctcacgcctgtaatcccagcactttgggaggccgaggcgggcggatcacgaggtcaggagatcgagaccatcctggctaacacggtgaaaccccgtctctactaaaaatacaaaaaattagccgggcgaggtggcgggcgcctgtagtcccagctactcgggaggctgaggcaggagaatggcgtgaacccgggaggcggagcttgcagtgagccgagatcgcgccactgcactccagcctgggcgacagcgagactccgtctc

>AluYf1

ggccgggcgcggtggctcacgcctgtaatcccagcactttgggaggccgaggcgggcggatcacgaggtcaggagatcgagaccatcctggctaacacggtgaaaccccgtctctactaaaaatacaaaaaattagccgggcgtggtggcgggcgcctgtagtcccagctacgcgggaggctgaggcaggagaatggcgtgaacccgggaggcggagcttgcagtgagccgagatcgcgccactgcactccagcctgggcgacagagcgagactccgtctc

>AluYf2

ggccgggcgcggtggctcacgcctgtaatcccagcactttgggaggccgaggcgggcggatcacgaggtcaggagatcgagaccatcctggctaacacagtgaaaccccgtctctactaaaaaacacaaaaaaattagccgggcgtggtggcgggcgcctgtagtcccagctacgcgggaggctgaggcaggagaatggcgtgaacccgggaggcggagcttgcagtgagccgagatcgcgccactgcactccagcctgggcgacagagcgagactccgtctc

>AluYf5

ggccgggcgcggtggctcacgcctgtaatcccagcactttgggaggccgaggcgggcggatcacgaggtcaggagatcgagaccatcctggctaacacggtgaaaccccgtctctactaaaaatacaaaaaattagccgggcgaggtggcgggcgcctgtagtcccagctacgcgggaggctgaggcaggagaatggcgtgaaccccggggggcggagcctgcagtgagccgagatcgcgccactgcactccagcctgggcgacagcgagactccgtctc

>AluYg6

ggccgggcgcggtggctcacgcctgtaatcccagcactttgggaggccgagacgggcggatcacgaggtcaggagatcgagaccatcctggctaacacggtgaaaccccgtctctactaaaaatacaaaaaattagccgggcatggtggcgcgcgcctgtagtcccagctacacgggaggctgaggcaggagaatggcgtgaacccgggaggcggagcttgcagtgagtcgagatcgcgccactgcactccagcctgggcgacagagcgaaactccgtctc

>AluYh9

ggccgggcgcggtggctcacgcctgtaatcccagcactttgggaggcagaggcgggcggatcatgaggtcaggagatcgagaccatcctggctaacgcggtgaaaccccgcctctactaaaaatacaaaaaattagccgggcgtggtggcgggcgcctgtggtcccggctactcgggaggctgaggcaggagaatggcgtgaacccgggaggcggagcttgcagtgagctgaggtcgcgccactgcaccccagcctgggcgacagagcgagactccgtctc

>AluYi6

ggccgggcgcggtggctcacgcttgtaatcccagcactttgggaggccgaggcgggcggatcacgaggtcaggagatcgagaccatcctggctaacacggtgaaaccccgtctctactaaaaatacaaaaaaattagccgggcgtgatggcgggcgcctgtagtcccagctactcgggaggctgaggcaggagaatggcgtgaacccgggaggcggagcttgcagtgagccgagattgcgccactgcactcccgcctgggccacagagcgagactccgtctc

>AluYk11

ggctgggcgcggtggctcacgcctgtaatcccagcactttgggaggccgaggcgggcggatcacaaggtcaggagatcgagaccatcttggctaacacggtgaaaccccgtctctactaaaaatacaaaaaattagccgggcgcggtggcgggcgcctgtagtcccagctactcgggaggctgaggcaggagaatggcgtgaacctgggaggcggagcttgcagtgagccgagattgcgccactgcaatccggcctgggctaaagagcgggactccgtctc

>AluYk12

ggccgggcgcggtggctcacgcctgtaatcccagcactttgggaggccgaggcgggcggatcacgaggtcaggagatcgagaccatcctggctaacacggtgaaaccccgtctctactaaaaatacaaaaaattagccgggcgtggtggtgggcgcctgtaatcccagctactcgggaggctgaggcaggagaatggcatgaacccaagaggcggagcttgcagtgagccgggatagcgccactgcagtccagcttgggcgaaagagtgagactccgtctc

>AluYk13

ggccgggcgtggtggctcacgcctgtaatcccagcactttgggaggccgaggcgggtggatcatgaggtcaggagatcgagaccatcctggctaacacagtgaaaccccgtctctactaaaaatacaaaaaattagccgggagcggtggcgggctcctgtagtcccagctacttgagaggctgaggcaggagaatggcgtgaacccaggaggcggagcttgcagtgagccgagatcgcgccactgcactccagcctgggcgacagagccagacgctgtctc

>AluYj3

GGCCGGGCGCGGTGGCTCACGCCTGTAATCCCAGCACTTTGGGAGGCCGAGGCGGGCGGATCACGAGGTCAGGAGATCGAGACCATCCTGGCTAACACGGTGAAACCCCGTCTCTACTAAAAATGCAAAAAATTAGCCGGGCGTGGTGGCGGGCGCCTGTAGTCCCAGCTGCTCGGGAGGCTGAGGCAGGAGAATGGCGTGAACCCGGGAGGCGGAGCTTGCAGTGAGCCGAGATCGTGCCACTGCACTCCAGCCTGGGCGACAGAGCGAGACTCCGTCTC

>AluYj4

GGCCGGGCGCGGTGGCTCGCGCCTGTAATCCCAGCACTTTGGGAGGCCGAGGCGGGCGGATCACGAGGTCAGGAGATCGAGACCATCCTGGCTAACACGGTGAAACCCCGTCTCTACTAAAAATGCAAAAAATTAGCCGGGCGTGGTGGCGGGCGCCTGTAGTCCCAGCTGCTCGGGAGGCTGAGGCAGGAGAATGGCGTGAACCCGGGAGGCGGAGCTTGCAGTGAGCCGAGATCGTGCCACTGCACTCCAGCCTGGGCGACAGAGCGAGACTCCGTCTC

>AluYh3a1

GGCCGGGCGCGGTGGCTCACGCCTGTAATCCCAGCACTTTGGGAGGCAGAGGCGGGCGGATCATGAGGTCAGGAGATCGAGACCATCCTGGCTAACACAGTGAAACCCCGCCTCTACTAAAAATACAAAAAATTAGCCGGGCGTGGTGGCGGGCGCCTGTAGTCCCAGCTACTCGGGAGGCTGAGGCAGGAGAATGGCGTGAACCCGGGAGGCGGAGCTTGCAGTGAGCCGAGATCGCGCCACTGCACTCCAGCCTGGGCGACAGAGCGAGACTCCGTCTC

>AluYh3a3

GGCCGGGCGCGGTGGCTCACGCCTGTAATCCCAGCACTTTGGGAGGCAGAGGCGGGCGGATCATGAGGTCAGGAGATCGAGACCATCCTGGCTAACACAGTGAAACCCCGCCTCTACTAAAAATACAAAAAATTAGCCAGGCGTGGTGGCGGGCGCCTGTAGTCCCAGCTACTCGGGAGGCTGAGGCAGGAGAATGGCGTGAACCCGGGAGGCGGAGCTTGCAGTGAGCCGAGATCCCGCCGACAGAGCGAGACTCCGTCTC

>AluYh7

GGCCGGGCGCGGTGGCTCACGCCTGTAATCCCAGCACTTTGGGAGGCAGAGGCGGGCGGATCATGAGGTCAGGAGATCGAGACCATCCTGGCTAACGCGGTGAAACCCCGCCTCTACTAAAAATACAAAAAATTAGCCGGGCGTGGTGGCGGGCGCCTGTGGTCCCGGCTACTCGGGAGGCTGAGGCAGGAGAATGGCGTGAACCCGGGAGGCGGAGCTTGCAGTGAGCCGAGGTCGCGCCACTGCACTCCAGCCTGGGCGACAGAGCGAGACTCCGTCTC

>AluYg6a2

GgccgggcgcggtggctcacgcctgtaatcccagcactttgggaggccgagacgggcggatcacgaggtcAggagatcgagaccatcctggctaacacggagaaaccccgtctctactaaaaatacaaaaaattagccgggcatGgtggcgcgtgcctgtagtcccagctacacaggaggctgaggcaggagaatggcgtgaacccgggaggcggagcttgcagtgagtcgagatcgcgccactgcactccagcctgggcgacagagcgaaactccgtctc

>AluYg5b3

GgccgggcgcggtggctcacgcctgtaatcccagcactttgggaggccgagacgggcggatcacgaggtcAggagatcgagaccatcctggctgacacggagaaaccccgtctctactaaaaatacaaaaaattagccgggcatGgtggcgagcgcctgtagtcccagctactcgggaggctgaggcaggagaatggcgtgaacccgggaggcggagcttgcagtgagtcgagatcgcgccactgcgctccagcctgggcgacagagcgaaactccgtctc

>AluYh3

GGCCGGGCGCGGTGGCTCACGCCTGTAATCCCAGCACTTTGGGAGGCAGAGGCGGGCGGATCATGAGGTCAGGAGATCGAGACCATCCTGGCTAACACGGTGAAACCCCGCCTCTACTAAAAATACAAAAAATTAGCCGGGCGTGGTGGCGGGCGCCTGTAGTCCCAGCTACTCGGGAGGCTGAGGCAGGAGAATGGCGTGAACCCGGGAGGCGGAGCTTGCAGTGAGCCGAGATCGCGCCACTGCACTCCAGCCTGGGCGACAGAGCGAGACTCCGTCTC

>AluYa5a2

GGCCGGGCGCGGTGGCTCACGCCTGTAATCCCAGCACTTTGGGAGGCCGAGGCGGGCGGATCACGAGGTCAAGAGATCGAGACCATCCCCGCTAAAACGGTGAAACCCCGTCTCTACTAAAAATACAAAAAAATTAGCCGGGCGTAGTGGCGGGCGCCTGTAGTCCCAGCTACTTGGGAGGCTGAGGCAGGAGAATGGCGTGAACCCGGGAGGCGGAGCTTGCAGTGAGCCGAGATCCCGCCACTGCACTCCAGCCTGGGCGACAGAGCGAGACTCCGTCTC

>AluYa5b2

GGCCGGGCGCGGTGGCTCACGCCTGTAATCCCAGCACTTTGGGAGGCCGAGGCGGGCGGATCACGAGGTCAGGAGATCGAGACCAACCCGGCTAAAACGGTGAAACCCCGTCTCTACTAAAAATACAAAAAATTAGCCGGGCGTAGTGGCGGGCGCCTGTAGTCCCAGCTACTTGGGAGGCTGAGGCAGGAGAATGGCGTGAACCCGGGAGGCGGAGCTTGCAGTGAGCCGAGATCCCGCCACTGCACTCCAGCCTGGGCGACAGAGCGAGACTCCGTCTC

>AluYa5a1

GGCCGGGCGCGGTGGCTCACGCCTGTAATCCCAGCACTTTGGGAGGCCGAGGCGGGCGGATCACGAGGTCAGGAGATCGAGGCCATCCCGGCTAAAACGGTGAAACCCCGTCTCTACTAAAAATACAAAAAATTAGCCGGGCGTAGTGGCGGGCGCCTGTAGTCCCAGCTACTTGGGAGGCTGAGGCAGGAGAATGGCGTGAACCCGGGAGGCGGAGCTTGCAGTGAGCCGAGATCCCGCCACTGCACTCCAGCCTGGGCGACAGAGCGAGACTCCGTCTC

>AluYa5b1

GGCCGGGCGCGGTGGCTCACGCCTGTAATCCCAGCACTTTGGGAGGCCGAGGCGGGCGGATCACGAGGTCAGGAGATCGAGACCATCCCGGCTAAAACGGTGAAACCCCGTCTCTACTAAAAATACAAAAAATTAGCCGGGCGTAGTGGCGGGCGCCTGTGGTCCCAGCTACTTGGGAGGCTGAGGCAGGAGAATGGCGTGAACCCGGGAGGCGGAGCTTGCAGTGAGCCGAGATCCCGCCACTGCACTCCAGCCTGGGCGACAGAGCGAGACTCCGTCTC

>AluYa2

GGCCGGGCGCGGTGGCTCACGCCTGTAATCCCAGCACTTTGGGAGGCCGAGGCGGGCGGATCACGAGGTCAGGAGATCGAGACCATCCTGGCTAACACGGTGAAACCCCGTCTCTACTAAAAATACAAAAAATTAGCCGGGCGTAGTGGCGGGCGCCTGTAGTCCCAGCTACTTGGGAGGCTGAGGCAGGAGAATGGCGTGAACCCGGGAGGCGGAGCTTGCAGTGAGCCGAGATCGCGCCACTGCACTCCAGCCTGGGCGACAGAGCGAGACTCCGTCTC

>AluYa3_1

GGCCGGGCGCGGTGGCTCACGCCTGTAATCCCAGCACTTTGGGAGGCCGAGGCGGGCGGATCACGAGGTCAGGAGATCGAGACCATCCCGGCTAAAACGGTGAAACCCCGTCTCTACTAAAAATACAAAAAATTAGCCGGGCGTAGTGGCGGGCGCCTGTAGTCCCAGCTACTCGGGAGGCTGAGGCAGGAGAATGGCGTGAACCCGGGAGGCGGAGCTTGCAGTGAGCCGAGATCGCGCCACTGCACTCCAGCCTGGGCGACAGAGCGAGACTCCGTCTC

>AluYb5

GGCCGGGCGCGGTGGCTCACGCCTGTAATCCCAGCACTTTGGGAGGCCGAGGCGGGTGGATCATGAGGTCAGGAGATCGAGACCATCCTGGCTAACAAGGTGAAACCCCGTCTCTACTAAAAATACAAAAAATTAGCCGGGCGCGGTGGCGGGCGCCTGTAGTCCCAGCTACTCGGGAGGCTGAGGCAGGAGAATGGCGTGAACCCGGGAGGCGGAGCTTGCAGTGAGCCGAGATTGCGCCACTGCACTCCAGCCTGGGCGACAGAGCGAGACTCCGTCTC

>AluYb6_1

GGCCGGGCGCGGTGGCTCACGCCTGTAATCCCAGCACTTTGGGAGGCCGAGGCGGGTGGATCATGAGGTCAGGAGATCGAGACCATCCTGGCTAACAAGGTGAAACCCCGTCTCTACTAAAAATACAAAAAATTAGCCGGGCGTGGTGGCGGGCGCCTGTAGTCCCAGCTACTCGGGAGGCTGAGGCAGGAGAATGGCGTGAACCCGGGAGGCGGAGCTTGCAGTGAGCCGAGATTGCGCCACTGCAGTCCGCAGTCCGGCCTGGGCGACAGAGCGAGACTCCGTCTC

>AluYb10

GGCCGGGCGCGGTGGCTCACGCCTGTAATCCCAGCACTTTGGGAGGCCGAGGCGGGTGGATCATGAGGTCAGGAGATCGAGACCATCCTGGCTAACAAGGTGAAACCCCGTCTCTACTAAAAATACAAAAAATTAGCCGGGCGCGGTGGCGGGCGCCTGTAGTCCCAGCTACTGGGGAGGCTGAGGCAGGAGAATGGCGTGAACCCGGGAAGCGGAGCTTGCAGTGAGCCGAGATTGCGCCACTGCAGTCCGCAGTCCAGCCTGGGCGACAGAGCGAGACTCCGTCTC

>AluYb11

GGCCGGGCGCGGTGGCTCACGCCTGTAATCCCAGCACTTTGGGAGGCCGAGGCGGGTGGATCATGAGGTCAGGAGATCGAGACCATCCTGGCTAACAAGGTGAAACCCCGTCTCTACTAAAAATACAAAAAATTAGCCGGGCGCGGTGGCGGGCGCCTGTAGTCCCAGCTACTGGGGAGGCTGAGGCAGGAGAATGGCGTTGAACCCGGGAAGCGGAGCTTGCAGTGAGCCGAGATTGCGCCACTGCAGTCCGCAGTCCAGCCTGGGCGACAGAGCGAGACTCCGTCTC

>AluYa3_2

GGCCGGGCGCGGTGGCTCACGCCTGTAATCCCAGCACTTTGGGAGGCCGAGGCGGGCGGATCACGAGGTCAGGAGATCGAGACCATCCCGGCTAACACGGTGAAACCCCGTCTCTACTAAAAATACAAAAAATTAGCCGGGCGTGGTGGCGGGCGCCTGTAGTCCCAGCTACTTGGGAGGCTGAGGCAGGAGAATGGCGTGAACCCGGGAGGCGGAGCTTGCAGTGAGCCGAGATCCCGCCACTGCACTCCAGCCTGGGCGACAGAGCGAGACTCCGTCTC

>AluYa3_3

GGCCGGGCGCGGTGGCTCACGCCTGTAATCCCAGCACTTTGGGAGGCCGAGGCGGGCGGATCACGAGGTCAGGAGATCGAGACCATCCCGGCTAAAACGGTGAAACCCCGTCTCTACTAAAAATACAAAAAATTAGCCGGGCGTGGTGGCGGGCGCCTGTAGTCCCAGCTACTCGGGAGGCTGAGGCAGGAGAATGGCGTGAACCCGGGAGGCGGAGCTTGCAGTGAGCCGAGATCCCGCCACTGCACTCCAGCCTGGGCGACAGAGCGAGACTCCGTCTC

>AluYa3_4

GGCCGGGCGCGGTGGCTCACGCCTGTAATCCCAGCACTTTGGGAGGCCGAGGCGGGCGGATCACGAGGTCAGGAGATCGAGACCATCCTGGCTAACACGGTGAAACCCCGTCTCTACTAAAAATACAAAAAATTAGCCGGGCGTAGTGGCGGGCGCCTGTAGTCCCAGCTACTTGGGAGGCTGAGGCAGGAGAATGGCGTGAACCCGGGAGGCGGAGCTTGCAGTGAGCCGAGATCCCGCCACTGCACTCCAGCCTGGGCGACAGAGCGAGACTCCGTCTC

>AluYa3_5

GGCCGGGCGCGGTGGCTCACGCCTGTAATCCCAGCACTTTGGGAGGCCGAGGCGGGCGGATCACGAGGTCAGGAGATCGAGACCATCCTGGCTAAAACGGTGAAACCCCGTCTCTACTAAAAATACAAAAAATTAGCCGGGCGTAGTGGCGGGCGCCTGTAGTCCCAGCTACTTGGGAGGCTGAGGCAGGAGAATGGCGTGAACCCGGGAGGCGGAGCTTGCAGTGAGCCGAGATCGCGCCACTGCACTCCAGCCTGGGCGACAGAGCGAGACTCCGTCTC

>AluYa4_1

GGCCGGGCGCGGTGGCTCACGCCTGTAATCCCAGCACTTTGGGAGGCCGAGGCGGGCGGATCACGAGGTCAGGAGATCGAGACCATCCCGGCTAACACGGTGAAACCCCGTCTCTACTAAAAATACAAAAAATTAGCCGGGCGTAGTGGCGGGCGCCTGTAGTCCCAGCTACTTGGGAGGCTGAGGCAGGAGAATGGCGTGAACCCGGGAGGCGGAGCTTGCAGTGAGCCGAGATCCCGCCACTGCACTCCAGCCTGGGCGACAGAGCGAGACTCCGTCTC

>AluYa4_2

GGCCGGGCGCGGTGGCTCACGCCTGTAATCCCAGCACTTTGGGAGGCCGAGGCGGGCGGATCACGAGGTCAGGAGATCGAGACCATCCCGGCTAAAACGGTGAAACCCCGTCTCTACTAAAAATACAAAAAATTAGCCGGGCGTAGTGGCGGGCGCCTGTAGTCCCAGCTACTCGGGAGGCTGAGGCAGGAGAATGGCGTGAACCCGGGAGGCGGAGCTTGCAGTGAGCCGAGATCCCGCCACTGCACTCCAGCCTGGGCGACAGAGCGAGACTCCGTCTC

>AluYa4_3

GGCCGGGCGCGGTGGCTCACGCCTGTAATCCCAGCACTTTGGGAGGCCGAGGCGGGCGGATCACGAGGTCAGGAGATCGAGACCATCCCGGCTAAAACGGTGAAACCCCGTCTCTACTAAAAATACAAAAAATTAGCCGGGCGTGGTGGCGGGCGCCTGTAGTCCCAGCTACTTGGGAGGCTGAGGCAGGAGAATGGCGTGAACCCGGGAGGCGGAGCTTGCAGTGAGCCGAGATCCCGCCACTGCACTCCAGCCTGGGCGACAGAGCGAGACTCCGTCTC

>AluYa4_4

GGCCGGGCGCGGTGGCTCACGCCTGTAATCCCAGCACTTTGGGAGGCCGAGGCGGGCGGATCACGAGGTCAGGAGATCGAGACCATCCTGGCTAAAACGGTGAAACCCCGTCTCTACTAAAAATACAAAAAATTAGCCGGGCGTAGTGGCGGGCGCCTGTAGTCCCAGCTACTTGGGAGGCTGAGGCAGGAGAATGGCGTGAACCCGGGAGGCGGAGCTTGCAGTGAGCCGAGATCCCGCCACTGCACTCCAGCCTGGGCGACAGAGCGAGACTCCGTCTC

>AluYa4_5

GGCCGGGCGCGGTGGCTCACGCCTGTAATCCCAGCACTTTGGGAGGCCGAGGCGGGCGGATCACGAGGTCAGGAGATCGAGACCATCCCGGCTAAAACGGTGAAACCCCGTCTCTACTAAAAATACAAAAAATTAGCCGGGCGTAGTGGCGGGCGCCTGTAGTCCCAGCTACTTGGGAGGCTGAGGCAGGAGAATGGCGTGAACCCGGGAGGCGGAGCTTGCAGTGAGCCGAGATCGCGCCACTGCACTCCAGCCTGGGCGACAGAGCGAGACTCCGTCTC

>AluYb6_2

GGCCGGGCGCGGTGGCTCACGCCTGTAATCCCAGCACTTTGGGAGGCCGAGGCGGGTGGATCATGAGGTCAGGAGATCGAGACCATCCTGGCTAACAAGGTGAAACCCCGTCTCTACTAAAAATACAAAAAATTAGCCGGGCGCGGTGGCGGGCGCCTGTAGTCCCAGCTACTCGGGAGGCTGAGGCAGGAGAATGGCGTGAACCCGGGAAGCGGAGCTTGCAGTGAGCCGAGATTGCGCCACTGCACTCCAGCCTGGGCGACAGAGCGAGACTCCGTCTC

>AluYe4

GGCCGGGCGCGGTGGCTCACGCCTGTAATCCCAGCACTTTGGGAGGCCGAGGCGGGCGGATCACGAGGTCAGGAGATCGAGACCATCCTGGCTAACACGGTGAAACCCCGTCTCTACTAAAAATACAAAAAATTAGCCGGGCGAGGTGGCGGGCGCCTGTAGTCCCAGCTACTCGGGAGGCTGAGGCAGGAGAATGGCGTGAACCCCGGGGGGCGGAGCCTGCAGTGAGCCGAGATCGCGCCACTGCACTCCAGCCTGGGCGACAGAGCGAGACTCCGTCTC

>AluYe6

GGCCGGGCGCGGTGGCTCACGCCTGTAATCCCAGCACTTTGGGAGGCCGAGGCGGGCGGATCACGAGGTCAGGAGATCGAGACCATCCTGGCTAACACGGTGAAACCCCGTCTCTACTAAAAATACAAAAAATTAGCCGGGCGAGGTGGCGGGCGCCTGTAGTCCCAGCTACTCGGGAGGCTGAGGCAGGAGAATGGCGTGAACCCCGGGGGGCGGAGCCTGCAGTGAGCCGAGATCGCGCCACTGCACTCCAACCTGGGCGACAGCGAGACTCCGTCTC

>AluYa1_1

GGCCGGGCGCGGTGGCTCACGCCTGTAATCCCAGCACTTTGGGAGGCCGAGGCGGGCGGATCACGAGGTCAGGAGATCGAGACCATCCTGGCTAACACGGTGAAACCCCGTCTCTACTAAAAATACAAAAAATTAGCCGGGCGTAGTGGCGGGCGCCTGTAGTCCCAGCTACTCGGGAGGCTGAGGCAGGAGAATGGCGTGAACCCGGGAGGCGGAGCTTGCAGTGAGCCGAGATCGCGCCACTGCACTCCAGCCTGGGCGACAGAGCGAGACTCCGTCTC

>AluYa1_2

GGCCGGGCGCGGTGGCTCACGCCTGTAATCCCAGCACTTTGGGAGGCCGAGGCGGGCGGATCACGAGGTCAGGAGATCGAGACCATCCTGGCTAACACGGTGAAACCCCGTCTCTACTAAAAATACAAAAAATTAGCCGGGCGTGGTGGCGGGCGCCTGTAGTCCCAGCTACTCGGGAGGCTGAGGCAGGAGAATGGCGTGAACCCGGGAGGCGGAGCTTGCAGTGAGCCGAGATCCCGCCACTGCACTCCAGCCTGGGCGACAGAGCGAGACTCCGTCTC

>AluYb7_1

GGCCGGGCGCGGTGGCTCACGCCTGTAATCCCAGCACTTTGGGAGGCCGAGGCGGGTGGATCATGAGGTCAGGAGATCGAGACCATCCTGGCTAACAAGGTGAAACCCCGTCTCTACTAAAAATACAAAAAATTAGCCGGGCGCGGTGGCGGGCGCCTGTAGTCCCAGCTACTCGGGAGGCTGAGGCAGGAGAATGGCGTGAACCCGGGAAGCGGAGCTTGCAGTGAGCCGAGATCGCGCCACTGCAGTCCGCAGTCCGGCCTGGGCGACAGAGCGAGACTCCGTCTC

>AluYb7_2

GGCCGGGCGCGGTGGCTCACGCCTGTAATCCCAGCACTTTGGGAGGCCGAGGCGGGTGGATCATGAGGTCAGGAGATCGAGACCATCCTGGCTAACAAGGTGAAACCCCGTCTCTACTAAAAATACAAAAAATTAGCCGGGCGTGGTGGCGGGCGCCTGTAGTCCCAGCTACTCGGGAGGCTGAGGCAGGAGAATGGCGTGAACCCGGGAAGCGGAGCTTGCAGTGAGCCGAGATTGCGCCACTGCAGTCCGCAGTCCGGCCTGGGCGACAGAGCGAGACTCCGTCTC

>AluYb7_3

GGCCGGGCGCGGTGGCTCACGCCTGTAATCCCAGCACTTTGGGAGGCCGAGGCGGGTGGATCACGAGGTCAGGAGATCGAGACCATCCTGGCTAACAAGGTGAAACCCCGTCTCTACTAAAAATACAAAAAATTAGCCGGGCGCGGTGGCGGGCGCCTGTAGTCCCAGCTACTCGGGAGGCTGAGGCAGGAGAATGGCGTGAACCCGGGAAGCGGAGCTTGCAGTGAGCCGAGATTGCGCCACTGCAGTCCGCAGTCCGGCCTGGGCGACAGAGCGAGACTCCGTCTC

>AluYb7_4

GGCCGGGCGCGGTGGCTCACGCCTGTAATCCCAGCACTTTGGGAGGCCGAGGCGGGTGGATCATGAGGTCAGGAGATCGAGACCATCCTGGCTAACAAGGTGAAACCCCGTCTCTACTAAAAATACAAAAAATTAGCCGGGCGCGGTGGCGGGCGCCTGTAGTCCCAGCTACTCGGGAGGCTGAGGCAGGAGAATGGCGTGAACCCGGGAGGCGGAGCTTGCAGTGAGCCGAGATTGCGCCACTGCAGTCCGCAGTCCGGCCTGGGCGACAGAGCGAGACTCCGTCTC

>AluYc2

ggccgggcgcggtggctcacgcctgtaatcccagcactttgggaggccgaggcgggcggatcacgaggtcaggagatcgagaccatcctggctaacaaggtgaaaccccgtctctactaaaaatacaaaaaattagccgggcgtggtagcgggcgcctgtagtcccagctactcgggaggctgaggcaggagaatggcgtgaacccgggaggcggagcttgcagtgagccgagatcgcgccactgcactccagcctgggcgacagagcgagactccgtctc

>AluYe5

Ggccgggcgcggtggctcacgcctgtaatcccagcactttgggaggccgaggcgggcggatcacgaggtcaggagatcgagaccatcctggctaacacggtgaaaccccgtctctactaaaaatacaaaaaattagccgggcgaggtggcgggcgcctgtagtcccagctactcgggaggctgaggcaggagaatggcgtgaaccccggggggcggagcctgcagtgagccgagatcgcgccactgcactccagcctgggcgacagcgagactccgtctc
